# Correction to “The Reflections of Global Climate Change on Wound and Ostomy Care: Awareness, Experiences, and Strategies in Nursing Practices”

**DOI:** 10.1111/iwj.70747

**Published:** 2025-08-12

T. Sengul, A. Ozakgul, D. Y. Akyaz, Y. Aydin, and D. Karakaya, “The Reflections of Global Climate Change on Wound and Ostomy Care: Awareness, Experiences, and Strategies in Nursing Practices,” *International Wound Journal* 22, no. 8 (2025): e70729, https://doi.org/10.1111/iwj.70729.

Reference citations have been adjusted and a new reference number [38] has been added.

38. T. Sengul, S. Sarikose, B. Uncu, and N. Kaya, “Predictive Role of Climate Change Awareness and Protective Behaviors on Quality of Life Among Nursing and Midwifery Students,” *Nurse Education Today* 154 (2025): 106831, https://doi.org/10.1016/j.nedt.2025.106831.

We apologize for this error.

